# Chinese translation and validation of the Near-Death Experience Content scale

**DOI:** 10.3389/fpsyt.2023.1201416

**Published:** 2024-01-10

**Authors:** Yan Li, Yan Chen, Charlotte Martial, Mingquan Shen, Héléna Cassol, Jing Yu, Xingyue Zhou, Chengcheng Ni, Meiqi Li, Nantu Hu, Olivia Gosseries, Steven Laureys, Haibo Di

**Affiliations:** ^1^International Vegetative State and Consciousness Science Institute, Hangzhou Normal University, Hangzhou, China; ^2^Coma Science Group, GIGA-Consciousness, University of Liège, Liège, Belgium; ^3^Centre du Cerveau^2^, University Hospital of Liège, Liège, Belgium; ^4^Joint International Research Unit on Consciousness, CERVO Brain Research Centre, Laval University, Québec, QC, Canada

**Keywords:** Near-Death Experience, scale, near-death-like experience, consciousness, phenomenology

## Abstract

**Introduction:**

In recent years, a growing number of near-death experience (NDE) testimonies have been collected worldwide due to an increasing interest in research on this phenomenon. China has many patients who survive life-threatening situations, leaving over much data on NDEs to be collected for research. In the historical context of Eastern civilization, many mentally controlled practices in China can also lead to “NDEs-like” (e.g., meditation). This study aimed (1) to translate and validate the recently developed Near-Death Experience Content (NDE-C) scale into Chinese and (2) to quantify and identify NDEs and NDEs-like in China with this new Chinese version of the NDE-C scale.

**Methods:**

Here, we presented the work that had been performed to translate the NDE-C scale into Chinese and validated this version on 79 NDE testimonies.

**Results:**

Brislin’s back-translation model was performed to translate a Chinese version of the NDE-C scale and internal consistency (the Cronbach’s α value for the total group = 0.846) as well as the confirmatory factor analysis was conducted.

**Discussion:**

Currently, the Chinese version of the NDE-C scale is ready for use in research practice in the context of Eastern culture, to screen people who have experienced an NDEs(-like) and to quantify their subjective experience, promoting further NDEs-related research in China.

## 1 Introduction

Near-death experiences (NDEs) are subjective experiences including prototypical self-related emotional and mystical features (1), which can be considered as an episode of disconnected consciousness (2). Out-of-body experiences (OBEs), the feeling of peace and well-being, altered time perception, seeing a bright light, seeing a tunnel, encountering people or spirits, and a sense of harmony and/or unity are among the typical NDE elements (3–8). Since 1975, when Moody (9) brought the term “NDE” into medical and bestselling literature, interest in NDEs has grown considerably. Since then, more and more NDE testimonies have been collected all around the world. NDEs are increasingly being described as a clear, identifiable physiological and psychological reality with clinical implications (3). NDE, with an incidence of about 20% in cardiac arrest survivors and 15% in patients surviving a protracted severe illness, is not a rare phenomenon (10–12). Currently, the phenomenon of NDEs can be divided into two categories: classical NDEs and near-death-like experiences (NDEs-like). Classical NDEs occurred in life-threatening situations, such as cardiac arrest, perioperative complications, electrocution, near-drowning, asphyxia, or attempted suicide (6, 11–13). On the other hand, it may also occur in non-life-threatening situations, or what are known as NDEs-like (14–19), including epilepsy (20), syncope (21), meditation (22), intense grief and anxiety (16), or drug use (23, 24).

In 2020, Martial et al. (2) suggested a unified framework for “consciousness” based on two original models (2, 25, 26), offering a novel perspective to the investigation of NDE. They considered consciousness as a multi-faceted concept, which has three main dimensions: wakefulness (i.e., eye opening), internal awareness (i.e., environmental stimulus-independent thoughts), and connectedness (i.e., connection to the environment). Classical NDEs are subjective experiences that dissociate the correlation of the three dimensions and can be viewed as inner awareness without contact with the environment and without wakefulness. NDEs-like are a more heterogeneous set of states with the possibility of different levels of wakefulness and connectedness in the presence of inner awareness. The most widespread explanations for the occurrence of NDEs tend to integrate psychological and neurobiological mechanisms (2, 12). Therefore, NDEs provide researchers with a unique perspective to better understand the neurological mechanisms of (disconnected) consciousness (2).

The Greyson scale is the most commonly used measure to assess NDEs (27). However, as elaborated in Martial et al. (28), this questionnaire has several significant limitations, such as not addressing negative NDEs, leading to many cases of overlooked negative NDEs (27, 29–32). As the interest in NDEs increased in recent years, the original scientific tool has no longer met the scientists’ needs, and it is increasingly important to accurately identify near-death experiencers (NDErs) to facilitate empirical research as well as facilitate reproducibility among evaluators. In this context, Martial and colleagues developed the Near-Death Experience Content (NDE-C) scale in 2020 to strictly screen the phenomenology of NDEs (28). They demonstrated that the newly developed scale has good psychometric properties, such as good internal consistency and concurrent validity. This scale notably reevaluated the composition of the NDE scale for negative NDEs. Meanwhile, it solved the problem that distressing NDEs were not quantified in the Greyson scales. Importantly, the NDE-C scale itself does not distinguish between classical NDEs and NDEs-like; It is intended to identify the content of NDEs independent of context. It is rather the context within which the subjective experience has been occurred that will allow for this distinction.

According to nearly 40 years of surveys, the majority of NDEs research has been done in North America and Western Europe, while much less has been done in Asia (33). In the historical background of traditional culture in the East, the exploration of consciousness issues has never left the attention of scholars and monks of traditional Eastern culture. In China, there are a lot of common mental control practices (e.g., meditation) happening in situations that are not life-threatening inducing “NDEs-like,” a series of strong subjective experiences close to the content of the NDE, which also suits for this scale. However, up until today, there has not been a scientific scale to identify and quantify NDEs-like in China. The use of the newly developed NDE-C scale in China may help researchers to better understand and interpret these complex and often life-changing experiences. Meanwhile, it could also be adopted to quantify conscious experiences with the different contexts in Eastern culture. Therefore, it is of great significance to translate and validate the recently developed NDE-C scale into Chinese (28). The official language of the translated scale is Mandarin.

## 2 Methods

### 2.1 The NDE-C scale: description and psychometric properties

The NDE-C scale is a self-reported instrument with 20 items associated with five factors: (1) Beyond the usual (NDE-C1, 2, 8, 9, 11, 20); (2) Harmony (NDE-C5 and 6); (3) Insight (NDE-C3, 4, 10, 13, 14); (4) Border (NDE-C12, 15, 16, 17, 18); (5) Gateway (NDE-C7, 19). This tool aims to identify the presence of an NDE phenomenology using a cut-off score of ≥27/80. Each item scores from 0 to 4 on a Likert-type scale (0 = “not at all; none,” 1 = “slightly,” 2 = “moderately,” 3 = “strongly; equivalent in degree to any other strong experience lived so far” and 4 = “extremely; more than any other time in my life and stronger than 3”). Items and scale scores are linearly transformed to a 0–80 total score with higher scores indicating richer subjective experience. The developers of the NDE-C scale demonstrated that the five-factor structure is employed to cover the main relevant aspects of NDE and that it has very good psychometric properties, including concurrent validity and very good internal consistency (28). These improvements have led to the use of the NDE-C scale in subsequent studies as a reliable scale to quantify the phenomenology of NDEs (28, 34).

### 2.2 Translation procedure

In order to translate a Chinese version of the NDE-C scale that strictly respects the structure and the content of the original version, we used the Brislin’s back-translation model (35). The model has been applied for cross-cultural translation, which includes forward translation, back translation, and linguistic adaptation.

First, we performed forward translation. A graduate student (YL) and a professor (HD) majoring in basic medicine, who are proficient in English, independently translated the English version into the Chinese version. A committee consisting of three Chinese native authors (YL, HD and XZ) and two psychological experts worked together to edit, review, and approve a first draft translation of the NDE-C scale.

Next, back translation and linguistic adaption were carried out. The first draft of the NDE-C scale was translated back into English by Abacus Consulting Services, a translation agency without knowledge of the English version. The translators and interpreters of the agency are certified by the state and federal courts of the United States. A separate committee, consisting of one original developer of the NDE-C (CM) and four authors (YL, HD, XZ, and JY), evaluated the back translation to discuss linguistic errors or ambiguities and revised the first draft of the Chinese translation accordingly. The committee compared the back translation with the original scale and repeated the above steps, trying to make the Chinese version of the scale closest to the original English scale in terms of meaning.

Then, we conducted a pilot study and distributed the Chinese version to 20 participants who claimed to have experienced NDEs to evaluate the items’ fluency and comprehensibility and to identify whether there existed any confusion or difficulty in comprehending each item. We made appropriate modifications according to their feedback. Given the taboo surrounding death in traditional Chinese society, we adjusted the scale’s title to some extent to decrease people’s potential reluctance to fill out the scale (36). The adapted title was “Experience Content Scale.” Finally, the Chinese version of the NDE-C scale was formed.

### 2.3 Validation procedure

#### 2.3.1 Participants

Seventy-nine native Mandarin-speaking participants who claimed to have experienced NDEs (i.e., NDErs) (including the 20 NDErs of the pilot study) were recruited through social media via the website.^1^ They were capable of clearly remembering an NDE, aged over 17 years old, and had no history of neurological or psychiatric disorders. They completed the Chinese translation of the NDE-C scale which included socio-demographic information (i.e., gender, interview age, religion, nation, education level) and clinical characteristics (i.e., precipitating factors, etiology). Data were collected from December 2021 to December 2022.

#### 2.3.2 Statistics

Content validity was used to determine whether the scale’s items could correctly and sufficiently evaluate the concept (37). A panel of five experts in medicine and psychology (not including the experts in translation procedure) examined the cultural and semantic equivalency as well as the content validity index (CVI) of the NDE-C Chinese version, as in Huang et al. (38). To evaluate the CVI at the item-level CVI (I-CVI), each item was graded on a 4-point Likert scale to rate the relevance between the Chinese version and the original version by each expert (1 = “not relevant,” 2 = “somewhat relevant,” 3 = “quite relevant,” and 4 = “highly relevant”) (39). The I-CVI was determined by calculating the number of experts who scored “3” or “4” at all items and then dividing by the total number of experts (i.e., 5) (39). If the value of I-CVI is 1, the content validity is considered good (39, 40).

The data was split into two categories based on the context of the clinical characteristics of the NDEs: classical NDEs and NDEs-like.

We performed descriptive statistics on the socio-demographic information. The Shapiro–Wilk test was used to test for normality across all variables (*p* < 0.05). All distributions were skewed except for the NDE-C scale’s total score. Data were reported as medians (inter-quartile range, IQR).

We conducted internal consistency analysis in the total NDEs group and the classical NDEs group. The scale’s internal consistency was assessed by Cronbach’s α and by the correlations of each item with the total score. Cronbach’s α has a minimum acceptable value of 0.70 and a maximum acceptable value of 0.90 (41). A value below 0.70 shows a low internal consistency of the scale, whereas a score larger than 0.90 suggests redundancy or duplication of items within the scale.

Confirmatory factor analysis was based on the best conceptual fit. Communalities were examined to access the scale factorability. To determine whether the original five-factor model was supported by the data, the 20-item instrument was evaluated using criteria fit statistics to assess whether the suggested models were consistent with the data (28).

All analyses were performed using SPSS 25.0 and Amos 25.0.

### 2.4 Ethics statement

The study protocols were approved by the Ethics Committee of Hangzhou Normal University [NO: (20230322-4)]. Completion of the scale was voluntary for participants. The study was conducted according to the World Medical Association’s Declaration of Helsinki.

## 3 Results

### 3.1 Translation and cross-cultural adaptation

The final version of the Chinese translation of the NDE-C scale is shown in Supplementary material A. We made a few wording modifications to maintain the original scale while using descriptions that were more in tune with Chinese culture and way of life. The second item, “thoughts” literally means “an idea or opinion” in Chinese. There is a Chinese semantic problem: The translated noun cannot be paired with “speed up.” So, we made a slight modification to this expression. Some changes were also made to item four’s “understanding everything”: Since “understanding” refers to understanding the content or meaning in Chinese, it is easy to misunderstand here. After inquiring CM and discussing it with the group members, we modified it appropriately. The rest of the changes were simple word order changes, not obvious to the original change. Finally, it was determined by the committee that each item was simple, understandable, and acceptable. When we presented the scale to Chinese NDEs-like experiencers, we altered the name of the scale from “the Near-Death Experience Content (NDE-C) Scale” to “Experience Content Scale” due to Chinese people’s potential negative attitude toward death. Table 1 displays the exact wording for the 20 items after the Chinese translation method.

**Table 1:** The final Chinese version of the Near-Death Experience Content (NDE-C) scale (English translation into Chinese).

NDE-C item		Sentence
NDE-C1	Time perception 时间感知	Your perception of time was altered 你对时间的感知改变了
NDE-C2	Speeded thoughts 思考加快	Your thoughts speeded up 你的思考速度加快了
NDE-C3	Voice 声音	You heard one or several voices which did not have any material incarnation 你听到了没有任何物质化身的一个或几个声音
NDE-C4	Understanding 顿悟感	You had the feeling of suddenly understanding everything about yourself, the others and/or the universe 你产生了对于自己、他人或宇宙顿悟的感觉
NDE-C5	Peacefulness/well-being 平静/幸福	You had a feeling of peace and/or well-being 你产生了平静和/或幸福的感觉
NDE-C6	Harmony/unity 和谐/统一	You felt a sense of harmony or unity, as if you belonged to a larger whole 你体会到了和谐或统一的感觉，仿佛你从属于一个更大的整体
NDE-C7	Bright light 亮光	You saw or felt surrounded by a bright light without any determined material origin 你看到或感觉到被一束没有任何确定物质来源的亮光包围着
NDE-C8	Unusual sensation 不同寻常的感觉	You experienced unusual sensations (sight, hearing, smell, touch and/or taste) 你体验到不同寻常的感觉(视觉、听觉、嗅觉、触觉和/或味觉)
NDE-C9	Extrasensory perception 超感觉感知	You were aware of things beyond what your senses can usually perceive 你觉知到了超出你的感觉通常能够感知到的事物
NDE-C10	Precognition 预知力	You gained insightful knowledge about the future 你获得了对未来的深刻认识
NDE-C11	Out-of-body experience 处于自己身体之外	You had the impression of being outside of, or separated from your own body 你有了处于自己身体之外，或与自己身体分离的印象
NDE-C12	Leaving the earthly world 离开尘世	You had the sensation of leaving the earthly world or of entering a new dimension and/or environment 你有了离开尘世或进入新的维度和/或环境的感觉
NDE-C13	Life review 生活回顾	You saw or relieved events from your past 你看到或放下了你过去的事件
NDE-C14	Encounter 遇见	You encountered a presence and/or an entity (who might be deceased) 你遇见了一个存在物和/或实体(它可能已消亡)
NDE-C15	Non-existence/void/fear 不存在/空虚/恐惧	You had a feeling of non-existence, of being in a total void, and/or of fear 你有了一种不存在、处于完全空虚和/或恐惧状态的感觉
NDE-C16	Border/point of no return 没有回头路的边界/地点	You came close to a border and/or point of no return 你走近到一个没有回头路的边界和/或地点
NDE-C17	Come back 从体验中回来	You made the decision, or were forced, to come back from the experience 你做出决定，或被迫，从这种体验中回来
NDE-C18	Dying 濒死感	You had the feeling of dying and/or being dead 你有濒死和/或处于死亡状态的感觉
NDE-C19	Gateway 通道	You saw or entered a gateway (for instance a tunnel or a door) 你看到或进入了一个通道(例如一条隧道或一扇门)
NDE-C20	Ineffability 不可言说	You sense that the experience cannot be described adequately in words 你感到这种体验不能用语言充分描述

### 3.2 Validation procedure

#### 3.2.1 Content validity

For the final NDE-C Chinese version, items were all scored “3” or “4” by all the five experts. Therefore, the I-CVI value is 1. As a result, the Chinese NDE-C scale has good content validity, and no items should be removed.

#### 3.2.2 Participant characteristics

Table 2 shows the demographic characteristics of the total NDEs group constituting the retrospective study cohort [*N* = 79; 38 males (48%); median age at interview (IQR) = 27 (18–55) years]. The classical NDEs group [*N* = 43, 21 males, median age (IQR) = 27 (18–55) years] could be divided according to the etiology of the coma: “anoxic” (e.g., cardiac arrest, near-drowning and gas poisoning, *N* = 15); “traumatic” (e.g., motor vehicle accident, falls, *N* = 10); “complications of surgery or childbirth” (*N* = 11) and “other” (i.e., non-traumatic events such as hemorrhage or pesticide poisoning, *N* = 7). The NDEs-like group [*N* = 36, 17 males, median age (IQR) = 24 (18–53) years] included NDE occurring following a non-life-threatening event such as during sleep (*N* = 15), syncope (*N* = 7), meditation (*N* = 4), drugs and alcohol consumption (*N* = 3), or other non-life-threatening situations (*N* = 7). The education levels of the total NDEs group were as follows: 16 graduated from senior high school or below, 6 completed technical secondary or tertiary education, 38 had bachelor’s degrees, and 10 had master’s degrees or above. In terms of religion and beliefs, 86% of participants were agnostic or atheist (86% in both classical NDEs and NDEs-like) while others were religious. The majority of the participants were Han nationality (90%). Table 2 shows the detailed characteristics of the participants.

**Table 2:** Socio-demographic and clinical characteristics of the study sample.

Demographics	Total NDEs group (*N* = 79)	Classical NDEs group (*N* = 43)	NDEs-like group (*N* = 36)
**Gender**			
Male	38 (48%)	21 (49%)	17 (47%)
Female	41 (52%)	22 (51%)	19 (53%)
Age at interview median (IQR)	27 (18–55)	27 (18–55)	24 (18–53)
**Religion background**			
Agnostic or atheist	68 (86%)	37 (86%)	31 (86%)
Religious belief	11 (14%)	6 (14%)	5 (14%)
**Nation**			
Han	71 (90%)	38 (88%)	33 (92%)
Other	8 (10%)	5 (12%)	3 (8%)
**Education level**			
Senior high school and below	16 (20%)	10 (23%)	6 (17%)
Technical secondary or tertiary school	15 (19%)	10 (23%)	5 (14%)
Bachelor degree	38 (48%)	20 (47%)	18 (50%)
Master degree and above	10 (13%)	3 (7%)	7 (19%)

#### 3.2.3 Item statistics and distributional properties

The Chinese NDE-C scale total score mean was 39 ± 16 (out of 80) for the total group, 38 ± 18 (out of 80) for the classical NDEs group, and 39 ± 14 (out of 80) for the NDEs-like group. Supplementary material B comprises the frequency of each response for all NDE-C scale multiple-choice items. No item was reported by any participant as having a value of 1 on the Likert scale.

#### 3.2.4 Internal consistency and item-total correlation

The entire standardized Cronbach’s α estimate for the Chinese NDE-C scale was 0.85 for the total group and 0.88 for the classical NDEs group, which is regarded as acceptable. The correlation between each item and the total score ranged from 0.28 to 0.61 for the total group and 0.19 to 0.70 for the classical NDEs group. The estimation of Cronbach’s α for the total NDEs group (after removing each item from the pool of items to assess the independent contribution of each item to the measurement error in the scale) ranged from 0.83 to 0.85 (see Supplementary material C for details). The Cronbach’s α for the classical NDEs ranged from 0.86 to 0.88 after deleting each item and thus achieved the recommended 0.70. This shows that the items are homogenous and interdependent for the construct they measure.

#### 3.2.5 Confirmatory factor analysis

We followed the 5-factor structure from its original version for verification (28). In the total group, fit statistics of this 5-factor model were: χ^2^ = 275.400, χ^2^/DF = 1.721, *p* < 0.001; root mean squared error of approximation (RMSEA) = 0.096; standardized root-mean-squared residual (SRMR) = 0.101; Tucker-Lewis Index (TLI) = 0.693; Comparative Fit Index (CFI) = 0.741. In the classical NDEs group, fit statistics of this 5-factor model were: χ^2^ = 294.015, χ^2^/DF = 1.838, *p* < 0.001; RMSEA = 0.141; SRMR = 0.127; TLI = 0.544; CFI = 0.616. In the NDEs-like group, fit statistics of this 5-factor model were: χ^2^ = 317.736, χ^2^/DF = 1.986, *p* < 0.001; RMSEA = 0.168; SRMR = 0.155; TLI = 0.293; CFI = 0.404. In the total group, the median score (see Supplementary material D for details) of the *Beyond the usual* subscale was 11 (out of 24), the median score of the *Harmony* subscale was 5 (out of 8); median score of the *Insight* subscale was 4 (out of 20); median score of the *Border* subscale was 9 (out of 20); and median score of the *Gateway* subscale was 3 (out of 8). In the classical NDEs group, the median score of the *Beyond the usual* subscale was 11 (out of 24), the median score of the *Harmony* subscale was 7 (out of 8); the median score of the *Insight* subscale was 7 (out of 20); median score of the *Border* subscale was 10 (out of 20); and median score of the *Gateway* subscale was 3 (out of 8). In the NDEs-like group, the median score of the *Beyond the usual* subscale was 14 (out of 24), the median score of the *Harmony* subscale was 4 (out of 8); the median score of the *Insight* subscale was 10 (out of 20); median score of the *Border* subscale was 8 (out of 20), and median score of the *Gateway* subscale was 2 (out of 8). The above results indicate that for the Chinese translation of the NDE-C scale, the 5-factor structure is still the best conceptual fit in the context of Eastern culture.

#### 3.2.6 Cut-off score

In the total group, we observed 60 participants (76%) with total scores ≥27/80. In the classical NDEs group, we observed 30 participants (70%) with total scores ≥27/80. In the NDEs-like group, we observed 30 participants (83%) with total scores ≥27/80.

## 4 Discussion

The first purpose of this study was to rigorously translate the original NDE-C scale from English into Chinese. The differences between the two independent translations were slight, owing primarily to differences in language order. The committee’s translation discussion went equally well. In order to adapt to linguistic and cultural differences in different locations where diverse dialects are spoken, cross-cultural adaptation was carried out appropriately, as in Zhang et al. (42).

The second purpose of the present study was to validate the NDE-C in Chinese NDErs. Its internal consistency is good (the Cronbach’s α value of the Chinese version of the NDE-C scale for the total group = 0.85, the Cronbach’s α value of classical NDEs group = 0.88). The Chinese version of the NDE-C scale maintains the original five-factor structure in the confirmatory factor analysis, as opposed to Greyson’s four-factor structure, which was inconsistent with some certain scale items. Compared with the traditional common tool NDE Scale by Greyson, all the items of the Chinese version of the NDE-C scale were worded affirmatively, instead of questions. Also, the Chinese version of the NDE-C scale added items related to negative emotions, which is more in line with the actual situation of NDE case studies (43). We here showed that the Chinese version of the NDE-C scale has good psychometric properties in our sample study and still adapts in an Eastern cultural context.

China is a vast country with a large population, many ethnic groups, and many dialects. In our study, 83% of the participants were agnostics or atheists which also indicates that atheism prevails in China. The same word may have different meanings, all of which may lead to misconceptions of translated concepts. Mandarin, China’s official language, is the language carrier of the Chinese translation of the NDE-C scale, which has the broadest reach and allows the scale to be promoted and utilized in all regions and hospitals in China as well as the Chinese-speaking areas to the greatest extent.

Although this study had important research implications, there are still some limitations. First of all, we have used the English version for Chinese translation and not the French version which is the original version of the NDE-C scale. Secondly, this is a relatively small sample. This is notably due to the specificity of this population, which limited our recruitment. Moreover, the participants were self-selected, therefore there was a potential selection bias, and they may not be representative of the general population. Future studies should study the psychometric properties of the Chinese NDE-C scale in a larger sample of NDEs(−like) experiencers. Another limitation is that we did not collect objective medical information regarding the presence of a life-threatening event.

## 5 Conclusion

The Chinese version of the NDE-C scale has been translated and validated in the present study, and is now accessible for use in actual research. This tool can be used to screen people who have experienced an NDEs(−like) and to quantify their subjective experience, thereby leading to promoting the growth of NDEs-related research in China. The Chinese translation of the NDE-C scale can be a valuable instrument for further empirical research on NDEs(−like) with Asian cultural backgrounds. Further investigations will nevertheless be needed to study its psychometric properties. Further research should also be conducted to determine how difficult it is to adopt and propagate Mandarin in a country with various dialects and traditional characters.

## Data availability statement

The original contributions presented in the study are included in the article/Supplementary material, further inquiries can be directed to the corresponding author.

## Author contributions

The protocol for this study was conceived and designed by YL, CM, HC, HD, JY, XZ, OG, and SL. YL, JY, CN, and XZ all made important contributions to data collection. Data processing and interpretation were done by YL and MS. YL, YC, CM, HC, XZ, JY, CN, ML, and NH all contributed to the preparation of the manuscript, including drafting and revision. All authors had full access to all the data in the study and had final authority on whether or not to submit it for publication. All authors contributed to the article and approved the submitted version.

## Footnotes

^1^https://www.wjx.cn/vm/eNIco6m.aspx#
