# Supplementary material for: Chinese translation and validation of the Near-Death Experience Content scale
Source: Front Psychiatry. 2024 Jan 10;14:1201416. doi: 10.3389/fpsyt.2023.1201416 (PMC10806053; doi:10.3389/fpsyt.2023.1201416)
Supplement: Supplementary file 1 [file Table_1.DOCX]

Supplementary Material

**Chinese translation and validation of the Near-Death Experience Content (NDE-C) scale**

**Yan Li^1+^, Yan Chen^1+^, Charlotte Martial****^2, 3^, Mingquan Shen^1^, Héléna Cassol^2^, Jing Yu^1^, Xingyue Zhou^1^, Chengcheng Ni^1^, Meiqi Li^1^, Nantu Hu^1^, Olivia Gosseries^2, 3^, Steven Laureys^2, 3, 4^, Haibo Di^1^ ***

^1^International Vegetative State and Consciousness Science Institute, Hangzhou Normal University, Hangzhou, Zhejiang, China

^2^Coma Science Group, GIGA-Consciousness, University of Liège, Liège, Belgium

^3^Centre du Cerveau², University Hospital of Liège, Liège, Belgium

^4^Joint International Research Unit on Consciousness, CERVO Brain Research Centre, Laval University, Québec, Canada

Yan Li^1+^ and Yan Chen^1+^ These authors contributed equally to this work and shared the first authorship

*** Correspondence:**Haibo Di, International Vegetative State and Consciousness Science Institute, Hangzhou Normal University, Hangzhou, Zhejiang, China.
haibodi@hznu.edu.cn

**Supplementary Material A**

**NDE-C量表**

*我们希望你根据你在体验时（不是体验前，也不是体验后）的感受和想法来选择最合适的答案回答以下20项陈述（仅允许陈述一个答案）。*

*每种体验或感觉的强度不同，这就是为什么我们希望你使用下文对每项陈述所描述的评定等级(1-4)来确定你体验的强度。相反，如果你没有体验到陈述中所描述的现象，请选择“0-完全没有；无”。如果你在体验过程中多次体验到了相同现象，请考虑用最显著的现象进行回答。*

*评定等级:*

*0 - 完全没有；无*

*1 - 轻微*

*2 - 适中*

*3 - 强烈；程度上等同于迄今生活中的任何其他强烈体验*

*4 - 极度强烈；超过我生命中任何其他时间并且比3强烈*

|  | **0** | **1** | **2** | **3** | **4** |
| --- | --- | --- | --- | --- | --- |
| 1. 你对时间的感知改变了 |  |  |  |  |  |
| 2. 你的思考速度加快了 |  |  |  |  |  |
| 3. 你听到了没有任何物质化身的一个或几个声音 |  |  |  |  |  |
| 4. 你产生了对于自己、他人或宇宙顿悟的感觉 |  |  |  |  |  |
| 5. 你产生了平静和/或幸福的感觉 |  |  |  |  |  |
| 6. 你体会到了和谐或统一的感觉，仿佛你从属于一个更大的整体 |  |  |  |  |  |
| 7. 你看到或感觉到被一束没有任何确定物质来源的亮光包围着 |  |  |  |  |  |
| 8. 你体验到不同寻常的感觉（视觉、听觉、嗅觉、触觉和/或味觉） |  |  |  |  |  |
| 9. 你觉知到了超出你的感觉通常能够感知到的事物 |  |  |  |  |  |
| 10.你获得了对未来的深刻认识 |  |  |  |  |  |
| 11.你有了处于自己身体之外，或与自己身体分离的印象 |  |  |  |  |  |
| 12.你有了离开尘世或进入新的维度和/或环境的感觉 |  |  |  |  |  |
| 13.你看到或放下了你过去的事件 |  |  |  |  |  |
| 14.你遇见了一个存在物和/或实体（它可能已消亡） |  |  |  |  |  |
| 15.你有了一种不存在、处于完全空虚和/或恐惧状态的感觉 |  |  |  |  |  |
| 16.你走近到一个没有回头路的边界和/或地点 |  |  |  |  |  |
| 17.你做出决定，或被迫，从这种体验中回来 |  |  |  |  |  |
| 18.你有濒死和/或处于死亡状态的感觉 |  |  |  |  |  |
| 19.你看到或进入了一个通道（例如一条隧道或一扇门） |  |  |  |  |  |
| 20.你感到这种体验不能用语言充分描述 |  |  |  |  |  |

| ***Group*** | **All participants** | | | | | | **Classical NDEs group** | | | | | | **NDEs-likes group** | | | | | |
| --- | --- | --- | --- | --- | --- | --- | --- | --- | --- | --- | --- | --- | --- | --- | --- | --- | --- | --- |
| **NDE-C scale item** | **Number of responses by score (%)** | | | | | **Median score (min-max)** | **Number of responses by score (%)** | | | | | **Median score (min-max)** | **Number of responses by score (%)** | | | | | **Median score (min-max)** |
|  | **0** | **1** | **2** | **3** | **4** |  | **0** | **1** | **2** | **3** | **4** |  | **0** | **1** | **2** | **3** | **4** |  |
| **NDE-C1** | 16 (20.3%) | 12 (15.2%) | 12 (15.2%) | 12 (15.2%) | 27 (34.2%) | 2(0-4) | 8 (18.6%) | 7 (16.3%) | 4 (9.3%) | 8 (18.6%) | 16 (37.2%) | 3(0-4) | 8(22.2%) | 5(13.9%) | 8(22.2%) | 4(11.1%) | 11(30.6%) | 2(0-4) |
| **NDE-C2** | 28 (35.4%) | 8 (10.1%) | 22(27.8%) | 9 (11.4%) | 12 (15.2%) | 1.5 (0-4) | 18 (41.9%) | 5 (11.6%) | 7 (16.3%) | 4 (9.3%) | 9 (20.9%) | 1(0-4) | 10(27.8%) | 3 (8.3%) | 15(41.7%) | 5 (13.9%) | 3 (8.3%) | 2(0-4) |
| **NDE-C3** | 43 (54.4%) | 9 (11.4%) | 5 (6.3%) | 10 (12.7%) | 12 (15.2%) | 0 (0-4) | 24 (55.8%) | 6 (14.0%) | 2 (4.6%) | 3 (7.0%) | 8 (18.6%) | 0(0-4) | 19 (52.8%) | 3(8.3%) | 3 (8.3%) | 7 (19.4%) | 4 (11.1%) | 0(0-4) |
| **NDE-C4** | 22(27.8%) | 11 (13.9%) | 17 (21.5%) | 12 (15.2%) | 17(21.5%) | 2 (0-4) | 11 (25.6%) | 7 (16.3%) | 9(20.9%) | 7(16.3%) | 9 (20.9%) | 2(0-4) | 11 (30.6%) | 4 (11.1%) | 8 (22.2%) | 5(13.9%) | 8 (22.2%) | 2(0-4) |
| **NDE-C5** | 23 (29.1%) | 7 (8.9%) | 14 (17.7%) | 13 (16.5%) | 22 (27.8%) | 2(0-4) | 9 (20.9%) | 6 (13.9%) | 7 (16.3%) | 8 (18.6%) | 13 (30.2%) | 2(0-4) | 14(38.9%) | 1(2.8%) | 7(19.4%) | 5(13.9%) | 9(25.0%) | 2(0-4) |
| **NDE-C6** | 33 (41.8%) | 5 (6.3%) | 14 (17.7%) | 8 (10.1%) | 19 (24.1%) | 2(0-4) | 19(44.2%) | 3(7.0%) | 9(20.9%) | 3 (7.0%) | 9 (20.9%) | 1(0-4) | 14(38.9%) | 2(5.6%) | 5(13.9%) | 5(13.9%) | 10(27.8%) | 2(0-4) |
| **NDE-C7** | 29(36.7%) | 9 (11.4%) | 17(21.5%) | 7 (8.9%) | 17 (21.5%) | 1.5(0-4) | 14 (32.6%) | 5 (11.6%) | 9(20.9%) | 4 (9.3%) | 11 (25.6%) | 2(0-4) | 15(41.7%) | 4(11.1%) | 8(22.2%) | 3(8.3%) | 6(16.7%) | 1(0-4) |
| **NDE-C8** | 14 (17.7%) | 11 (13.9%) | 13 (16.5%) | 10 (12.7%) | 31 (39.2%) | 2(0-4) | 9(20.9%) | 7 (16.3%) | 6(14.0%) | 3 (7.0%) | 18 (41.9%) | 2(0-4) | 5(13.9%) | 4(11.1%) | 7(19.4%) | 7(19.4%) | 13(36.1%) | 3(0-4) |
| **NDE-C9** | 17(21.5%) | 8 (10.1%) | 14 (17.7%) | 14 (17.7%) | 26 (32.9%) | 2(0-4) | 12 (27.9%) | 6 (14.0%) | 6 (13.9%) | 4 (9.3%) | 15 (34.9%) | 2(0-4) | 5 (13.9%) | 2(5.6%) | 8(22.2%) | 10(27.8%) | 11(30.6%) | 3(0-4) |
| **NDE-C10** | 34(43.0%) | 9 (11.4%) | 13 (16.5%) | 10 (12.7%) | 13 (16.5%) | 1(0-4) | 20(46.5%) | 5(11.6%) | 7 (16.3%) | 5 (11.6%) | 6 (14.0%) | 1(0-4) | 14(38.9%) | 4(11.1%) | 6(16.7%) | 5(13.9%) | 7(19.4%) | 1.5(0-4) |
| **NDE-C11** | 18 (22.8%) | 10 (12.7%) | 10 (12.7%) | 13(16.5%) | 28 (35.4%) | 2(0-4) | 10 (23.3%) | 5 (11.6%) | 4(9.3%) | 6(13.9%) | 18 (41.9%) | 3(0-4) | 8(22.2%) | 5(13.9%) | 6(16.7%) | 7(19.4%) | 10(27.8%) | 2(0-4) |
| **NDE-C12** | 18 (22.8%) | 10 (12.7%) | 11 (13.9%) | 12 (15.2%) | 28 (35.4%) | 2(0-4) | 12 (27.9%) | 5(11.6%) | 7 (16.3%) | 3 (7.0%) | 16 (37.2%) | 2(0-4) | 6(16.7%) | 5(13.9%) | 4(11.1%) | 9(25.0%) | 12(33.3%) | 3(0-4) |
| **NDE-C13** | 33 (41.8%) | 9 (11.4%) | 9 (11.4%) | 7 (8.9%) | 21 (26.6%) | 1(0-4) | 17(39.5%) | 5(11.6%) | 5(11.6%) | 4 (9.3%) | 12 (27.9%) | 1(0-4) | 16(44.4%) | 4(11.1%) | 4(11.1%) | 3(8.3%) | 9(25.0%) | 1(0-4) |
| **NDE-C14** | 42 (53.2%) | 6(7.6%) | 9(11.4%) | 7(8.9%) | 15 (19.0%) | 0(0-4) | 24 (55.8%) | 3 (7.0%) | 6(14.0%) | 2 (4.6%) | 8(18.6%) | 0(0-4) | 18(50.0%) | 3(8.3%) | 3(8.3%) | 5(13.9%) | 7(19.4%) | 0.5(0-4) |
| **NDE-C15** | 17 (21.5%) | 7(8.9%) | 12(15.2%) | 14(17.7%) | 29 (36.7%) | 3(0-4) | 9 (20.9%) | 5(11.6%) | 5 (11.6%) | 7(16.3%) | 17(39.5%) | 3(0-4) | 8(22.2%) | 2(5.6%) | 7(19.4%) | 7(19.4%) | 12(33.3%) | 3(0-4) |
| **NDE-C16** | 37 (46.8%) | 4 (5.1%) | 11 (13.9%) | 5(6.3%) | 22 (27.8%) | 0(0-4) | 22 (51.2%) | 2 (4.6%) | 4 (9.3%) | 3 (7.0%) | 12 (27.9%) | 0(0-4) | 15(41.7%) | 2(5.6%) | 7(19.4%) | 2(5.6%) | 10(27.8%) | 2(0-4) |
| **NDE-C17** | 18(22.8%) | 8 (10.1%) | 19 (24.1%) | 8 (10.1%) | 26 (32.9%) | 2(0-4) | 10 (23.3%) | 4(9.3%) | 10 (23.3%) | 5(11.6%) | 14 (32.6%) | 2(0-4) | 8(22.2%) | 4(11.1%) | 9(25.0%) | 3(8.3%) | 12(33.3%) | 2(0-4) |
| **NDE-C18** | 16(20.3%) | 9 (11.4%) | 16 (20.3%) | 16 (20.3%) | 22 (27.8%) | 2(0-4) | 8 (18.6%) | 6 (13.9%) | 8 (18.6%) | 8 (18.6%) | 13(30.2%) | 2(0-4) | 8(22.2%) | 3(8.3%) | 8(22.2%) | 8(22.2%) | 9(25.0%) | 2(0-4) |
| **NDE-C19** | 40 (50.6%) | 7 (8.9%) | 7 (8.9%) | 2 (2.5%) | 23 (29.1%) | 0(0-4) | 24 (55.8%) | 2 (4.7%) | 3 (7.0%) | 1 (2.3%) | 13 (30.2%) | 0(0-4) | 16(44.4%) | 5(13.9%) | 4(11.1%) | 1(2.8%) | 10(27.8%) | 1(0-4) |
| **NDE-C20** | 6 (7.6%) | 9 (11.4%) | 21 (26.6%) | 15 (19.0%) | 28 (35.4%) | 3(0-4) | 5 (11.6%) | 5 (11.6%) | 12 (27.9%) | 6 (14.0%) | 15(34.9%) | 2(0-4) | 1(2.8%) | 4(11.1%) | 9(25.0%) | 9(25.0%) | 13(36.1%) | 3(0-4) |

**Supplementary Material B**

Response frequency distributions on the NDE-C scale for the classical NDEs and NDEs-Likes groups

**Supplementary Material C**

| NDE-C scale item | Correlation with NDE total score | α-value |
| --- | --- | --- |
| NDE1 | 0.287 | 0.845 |
| NDE2 | 0.365 | 0.841 |
| NDE3 | 0.345 | 0.842 |
| NDE4 | 0.522 | 0.835 |
| NDE5 | 0.359 | 0.842 |
| NDE6 | 0.532 | 0.834 |
| NDE7 | 0.322 | 0.843 |
| NDE8 | 0.386 | 0.840 |
| NDE9 | 0.568 | 0.833 |
| NDE10 | 0.412 | 0.839 |
| NDE11 | 0.462 | 0.837 |
| NDE12 | 0.611 | 0.830 |
| NDE13 | 0.396 | 0.840 |
| NDE14 | 0.576 | 0.832 |
| NDE15 | 0.381 | 0.841 |
| NDE16  NDE17  NDE18  NDE19  NDE20 | 0.498  0.363  0.350  0.306  0.493 | 0.835  0.841  0.842  0.845  0.837 |

Standardized variables of the principal component factor analysis on the NDE-C scale (N=79).

**Supplementary Material D**

| **Group** | **Beyond the usual**  **(Full Score=24）** | **Harmony**  **（Full Score=8）** | **Insight**  **（Full Score=20）** | **Border**  **（Full Score=20）** | **Gateway**  **（Full Score=8）** |
| --- | --- | --- | --- | --- | --- |
| **All participants** | 11 | 5 | 4 | 9 | 3 |
| **Classical NDEs** | 11 | 7 | 7 | 10 | 3 |
| **NDEs-like** | 14 | 4 | 10 | 8 | 2 |

median score for all participants, classical NDEs and NDEs-Likes groups in each factor
